# A Systematic Review of the Use and Quality of Qualitative Methods in Concept Elicitation for Measures with Children and Young People

**DOI:** 10.1007/s40271-020-00414-x

**Published:** 2020-04-29

**Authors:** Samantha Husbands, Paul Mark Mitchell, Joanna Coast

**Affiliations:** Health Economics Bristol, Population Health Sciences, Bristol Medical School, University of Bristol, 1-5 Whiteladies Road, Bristol, BS8 1NU UK

## Abstract

**Background:**

Qualitative research is recommended in concept elicitation for patient-reported outcome measures to ensure item content validity, and those developing measures are encouraged to report qualitative methods in detail. However, in measure development for children and young people, direct research can be challenging due to problems with engagement and communication.

**Objectives:**

The aim of this systematic review was to (i) explore the qualitative and adapted data collection techniques that research teams have used with children and young people to generate items in existing measures and (ii) assess the quality of qualitative reporting.

**Methods:**

Three electronic databases were searched with forward citation and reference list searching of key papers. Papers included in the review were empirical studies documenting qualitative concept elicitation with children and young people. Data on qualitative methods were extracted, and all studies were checked against a qualitative reporting checklist.

**Results:**

A total of 37 studies were included. The quality of reporting of qualitative approaches for item generation was low, with information missing on sampling, data analysis and the research team, all of which are key to facilitating judgements around measure content validity. Few papers reported adapting methods to be more suitable for children and young people, potentially missing opportunities to more meaningfully engage children in concept elicitation work.

**Conclusions:**

Research teams should ensure that they are documenting detailed and transparent processes for concept elicitation. Guidelines are currently lacking in the development and reporting of item generation for children, with this being an important area for future research.

**Electronic supplementary material:**

The online version of this article (10.1007/s40271-020-00414-x) contains supplementary material, which is available to authorized users.

## Key Points for Decision Makers

**Table:** 

The use of qualitative research for concept elicitation is important to ensuring the content validity of patient-reported outcome measures.
The quality of the reporting of qualitative concept elicitation for child and young person measures was generally poor, making judgements around the content validity of measure items challenging.
Few measures reported adapting their data collection techniques to be more suitable for children and young people, potentially missing opportunities to more meaningfully engage this population in item development, particularly younger children.
Those developing measures for children and young people would benefit from clear guidelines on how to undertake and report qualitative methods for concept elicitation.

## Introduction

The process of healthcare decision making, specifically measuring and comparing the clinical and cost effectiveness of healthcare technologies, interventions or services, can be facilitated through the development and use of patient-reported outcome measures (PROMs). PROMs are questionnaires designed to capture the clinical and broader outcomes of treatments from the perspectives of patients [1]. They comprise items that should be designed to represent the concepts and outcomes most important to the population in which a measure will be used. Empirical work to develop measure items will be referred to here as ‘concept elicitation’ [2] but can also be known as conceptual attribute development [3–5]. Patients are asked to complete PROMs before and after receiving an intervention to record any differences in their outcomes as a result. The focus of a measure’s items will vary according to whether a measure has been developed for use in a specific disease area (condition-specific) or for generic use, with the latter facilitating the comparison of patient outcomes across a broad range of health and social care conditions [1].

An important consideration for all PROMs is to ensure that the contained items are relevant and sensitive to changes in aspects such as the health or well-being of that population [6]. Guidance on PROM development from the US Food and Drug Administration (FDA) [5] and the International Society for Pharmacoeconomics and Outcomes Research (ISPOR) task force [7] suggests that qualitative, empirical research with the target population is essential to establishing a measure’s content validity, that is, whether it adequately captures the items of interest [8]. The goal of qualitative research is typically to understand a phenomenon from the perspectives of those who are knowledgeable, experienced or involved [9], and qualitative data are most commonly generated through listening to the views and experiences of participants. The FDA emphasise the importance of reaching data saturation for items, that is, ensuring that they achieve full coverage of all aspects important to a population and decision-making context. The importance of clear reporting of the qualitative development of these measures is also emphasised (e.g., [5, 10]) to allow users (i.e., clinicians, researchers, decision makers etc.) to decide on a measure’s content validity and how suitable it is for use.

The FDA give specific advice on PROM development in children and adolescents, centred around content validity and ensuring that measures can be understood and completed by children and young people (CYP) [10]. However, direct research with CYP can prove challenging for PROMs development [11]. This is because traditional qualitative methods are typically very adult-orientated and less appropriate for use with children, particularly with young children and those not able to articulate their opinions using formal or language-based methods [12–14]. Arbuckle and Abetz-Webb [11] suggest that further challenges include engaging children in research activities and finding methods that are appropriate to meet the different age and developmental abilities of CYP. Rowen and colleagues (2020) note similar issues with asking CYP to provide values for items for preference-based measures, with concerns around their understanding and ability to address the complexity of elicitation tasks [15]. This raises questions around whether and how researchers are developing items for PROMs with the CYP population, including how they are overcoming issues with involving CYP in direct research and how they are ensuring the generation of sensitive and valid measures.

This paper presents a systematic review of empirical studies documenting the development of measures using qualitative methods with CYP. The review has two aims: (i) to explore the qualitative methods that research teams have used with CYP to develop measure items, and whether methods have been adapted to suit the age and developmental needs of the population; and (ii) to explore the quality of the reporting of these methods. The discussion section of the paper synthesises the main findings from the retrieved studies and makes comparisons between what is being carried out in practice and the limited guidance available on CYP PROM development, as well as reporting standards in qualitative research generally.

## Methods

### Search Strategies for Studies

With a focus on exploring the qualitative approaches taken with CYP for concept elicitation, the search was designed to retrieve a breadth of papers, including condition-specific and generic measures. The search combined electronic database searching, reference list and forward citation searching of key papers and using existing systematic reviews of CYP measures to identify whether any of the measures featured had reported the use of qualitative methods in item development [16–18].

Three relevant electronic databases were searched: PubMed (includes MEDLINE), EMBASE and EconLit, with no limits on dates. The search was updated in November 2019. Search terms were developed in PubMed and adapted slightly to maximise sensitivity within each database. The terms used combined the population of interest (children and young people) with variations on the possible focus and outcomes of the developed measures (i.e., an economic, quality-of-life or well-being focus), with alternative terms for the methodological approach taken to measure development, centred around the language used in the FDA PROM development guidance (i.e., qualitative, qualitative research). The search terms developed for use in the electronic databases are detailed in Appendix 1 (see electronic supplementary material [ESM]). The ‘find citing articles’ feature of electronic journals was used to identify other studies that had cited key papers. Key papers for forward citation and reference list searching were studies that included a higher level of detail on the qualitative methods for item development, in anticipation that other papers may have followed and cited their work [19–24].

### Selection Method

The lead reviewer (SH) screened the title and abstracts of each paper identified through the search. If the abstract did not contain enough information to make a judgement on its relevance, the full-text version of the paper was downloaded. All duplicate articles were excluded. An independent reviewer (PMM) screened a proportion (5%) of all paper abstracts in one electronic database (PubMed) against the inclusion and exclusion study criteria to ensure agreement and consistency in the papers included. The independent screening of the abstracts encouraged the authors to clarify which studies were and were not considered relevant against the inclusion and exclusion criteria.

### Study Inclusion and Exclusion Criteria

Studies were included in the review if they were (i) empirical studies documenting the development of the items of a measure using qualitative research with CYP and (ii) were developing a measure for use with CYP aged between 0 and 18 years. Excluded studies included non-English language articles, review articles, methodological guidelines and research protocols. Studies were excluded if they only reported using qualitative methods for validation of items (rather than development) or if they only briefly cited or discussed linked and already existing/published item development work—although any linked articles were then searched (via Google Scholar) for possible inclusion in the review. Excluded studies extended to those that were found to be superseded by papers with more detail available on the qualitative concept elicitation work, if existing papers focused on the development of the same measure and no information important to the review was sacrificed. Finally, studies were excluded if they also involved those over the age of 18 years or if the qualitative research was undertaken with parents/guardians or families only, that is, no CYP were directly involved in the concept elicitation.

### Data Extraction and Quality of Reporting of Qualitative Methods

Data were extracted from each article into a data extraction form (see Appendix 2 [in ESM]) to ensure that the same information was captured for all studies [25]. Details recorded for all articles were the author(s) and paper characteristics (i.e., year, title and paper objective). Information was also recorded on the measure name, the type of measure (i.e., condition-specific, generic), the age of the CYP the measure was developed for and whether parents/guardians had been involved in development work. Information was documented on the qualitative methods used and studies were assessed for quality using principles from the 32-item ‘Consolidated criteria for reporting qualitative research (COREQ)’ tool [26], which focuses on the adequacy of reporting provided on the research team and reflexivity (i.e., reflections on how a researcher’s personal and professional biases may affect research processes and outcomes [27]), study design and the analysis of findings. Details on the qualitative research in the data extraction form was collected under the following headings: information available on sampling, qualitative methods used, approach to analysis and positive and negative reflections on the methods (both the authors’ and the reviewer’s [SH]). The form also collected details on whether any other methods were used (aside from qualitative) to develop the items. Data extraction was completed independently by a second author for 20% of publications, as was the quality check through the COREQ checklist (PMM).

### Synthesis of Results

Microsoft Excel was used to tabulate the extracted data. The data were then summarised and collated into a narrative report to describe the findings. After a summary of the paper characteristics, information from the articles were synthesised under two themes: (i) an overview of the qualitative approach used in CYP concept elicitation and (ii) the quality of reporting in concept elicitation for CYP.

## Results

### Search Results

**Fig. 1:**
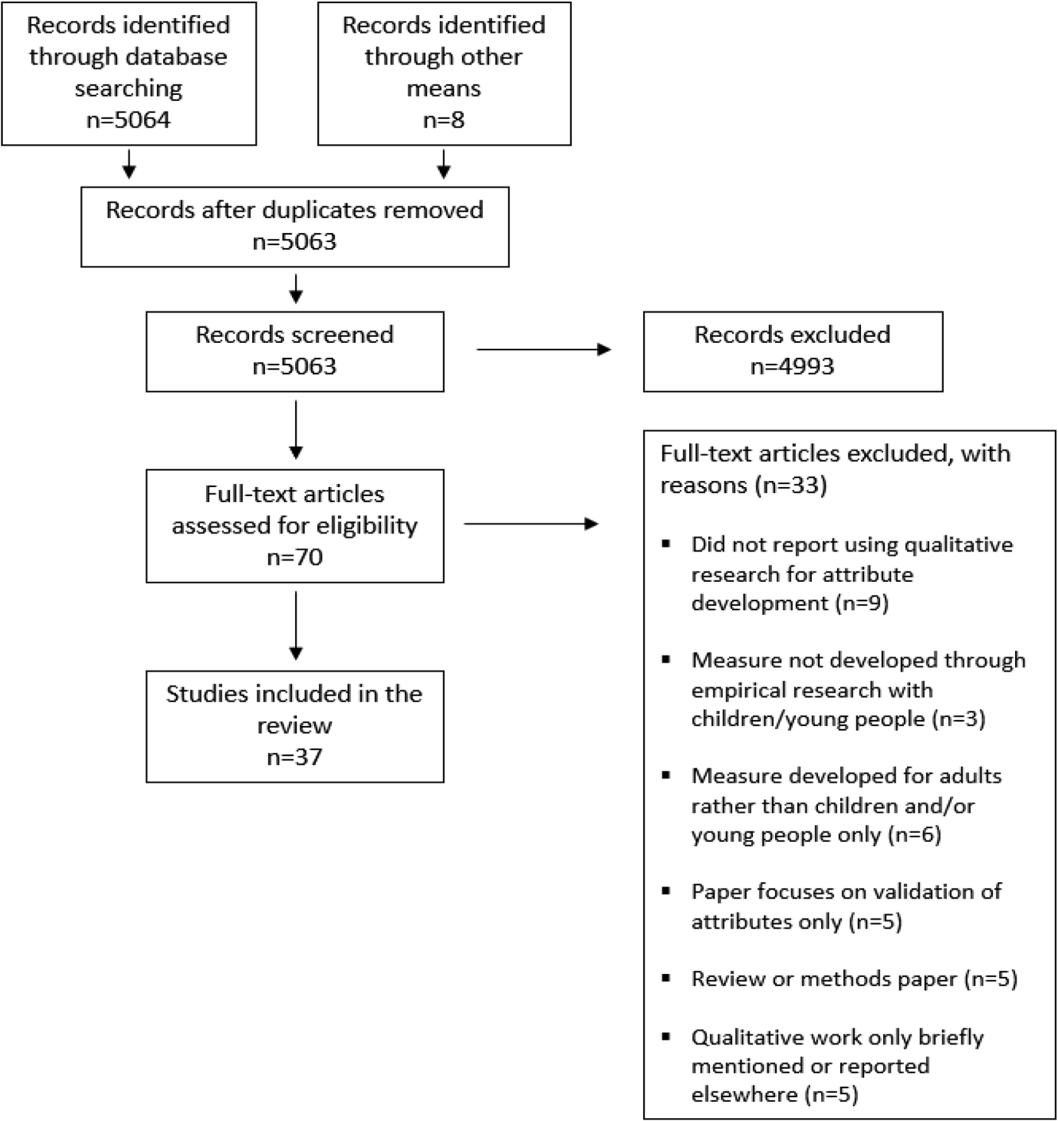
Study PRISMA flow diagram describing article selection procedure, Moher et al. [64]

The search strategy retrieved 5072 papers; nine duplicates were removed. After screening article abstracts and titles and full-text versions of the 70 articles retrieved, a total of 37 studies met the inclusion criteria and were included in the review. Of these, 29 were identified through electronic databases and eight through other means. One study retrieved in the review [28] was found to have a ‘sister’ paper that contained additional detail on the qualitative item development work but predated any specific CYP measure development [29]. Information from both studies were used to inform the review, but for clarity, were treated as one record [28]. The search process is documented in Fig. 1 and the full paper characteristics for the included papers are in Table 1. The result of the independent review of a proportion of all abstracts screened (*n* = 251) by two reviewers was an agreement of 99.6% abstracts to include/exclude (kappa statistic inter-rater agreement of 0.67, rated as ‘good’ [30]). There was no disagreement between SH and PMM regarding the accuracy and completeness of data extracted in the selected proportion of papers, including completion of the COREQ checklists.

**Table 1:** Retrieved paper characteristics

Authors	Paper name	Year	Name of measure	Aim(s) of the paper	Generic or condition-specific?	Age of children for which measure developed
Angeles-Han et al. [65]	Development of a vision related quality of life instrument for children 8–18 years of age for use in juvenile idiopathic arthritis-associated uveitis	2011	EYE-Q	To develop an instrument to assess the impact of juvenile idiopathic arthritis-associated uveitis on quality of life and evaluate the performance of activities that rely on vision in the home and school. The objective of this paper was to provide further evidence of the validity and reliability of the instrument (Effects of Youngsters' Eyesight on QOL (EYE-Q)) for children aged 8–18 years old	Condition-specific: juvenile idiopathic arthritis-associated uveitis	8–18 years
Basra et al. [66]	Conceptualisation, development and validation of T-QoL (Teenagers’ Quality of Life): a patient-focused measure to assess quality of life of adolescents with skin diseases	2017	T-QoL (Teenagers’ Quality of Life)	To use information directly from adolescents to give a comprehensive insight into the impact of skin diseases on their quality of life to develop an adolescent-specific instrument	Condition-specific: skin diseases	12–19 years
Beusterien et al. [32]	Development of the multi-attribute adolescent health utility measure (AHUM)	2012	Adolescent Health Utility Measure (AHUM)	To develop a multi-attribute measure that focuses on key impacts of treatment for chronic conditions among older children and adolescents	Condition-specific: chronic conditions	12–18 years
Bray et al. [20]	Defining health-related quality of life for young wheelchair users—a qualitative health economics study	2017	No abbreviated name mentioned	To explore how children with impaired mobility and their families define health-related quality of life and mobility-related quality of life in relation to wheelchair use and mobility impairment	Condition-specific: wheelchair use	0–18 years
Bruce et al. [42]	Development and preliminary validation of the KIDCLOT PAC QL: a new health-related quality-of-life measure for paediatric long-term anticoagulation therapy	2010	KIDCLOT PAC QL	To develop and initially validate a health-related quality-of-life inventory for children and their parents on long-term anticoagulation. The secondary objective was to determine features of long-term anticoagulation therapy that disrupt children’s and families’ health-related quality of life	Condition-specific: anticoagulation therapy	1–8 years
Das et al. [67]	Formation and psychometric evaluation of a health-related quality-of-life instrument for children living with HIV in India	2018	QOL-CHAI	To develop a culturally appropriate tool to assess the health-related quality of life to identify the areas of concern among the paediatric HIV population	Condition-specific: HIV	8–15 years
Davis et al. [50]	Quality of life of adolescents with cerebral palsy: perspectives of adolescents and parents	2008	CP QOL–Child.12	To use qualitative techniques to identify the important facets and domains of quality of life for adolescents with cerebral palsy	Condition specific: cerebral palsy	13–18 years
Flokstra-de Blok et al. [68]	Development and validation of the self-administered Food Allergy Quality of Life Questionnaire for adolescents	2008	FAQLQ-TF	The paper reports work on the development and cross-sectional validation of the first self-administered, food-allergy-specific, health-related quality-of-life questionnaire for adolescents: The Food Allergy Quality of Life Questionnaire–Teenager Form (FAQLQ-TF)	Condition-specific: food allergy	13–17 years
Fiume et al. [44]	Development and validation of the Paediatric Stroke Quality of Life Measure	2018	Paediatric Stroke Quality of Life Measure (PSQLM)	The paper reports on the development and validation of the Paediatric Stroke Quality of Life Measure (PSQLM), a novel instrument for measuring the quality of life of children after stroke	Condition-specific: stroke	2–18 years
Follansbee-Junger et al. [43]	Development of the PedsQL™ epilepsy module: focus group and cognitive interviews	2016	PedsQL Epilepsy Module	To create an epilepsy specific module of the PedsQL. The purpose of this paper was to describe the first three steps of the validation process, including how the items were generated, modified and adapted	Condition-specific: epilepsy	2–18 years
Franciosi et al. [38]	Quality of life in pediatric eosinophilic esophagitis: What is important to patients?	2012	No abbreviated name mentioned	To conduct focus interviews of paediatric patients with eosinophilic esophagitis and their parents to identify the key eosinophilic esophagitis disease-specific health-related quality of life concerns	Condition-specific: eosinophilic esophagitis	2–18 years
Geister et al. [69]	Qualitative development of the ‘Questionnaire on Pain caused by Spasticity (QPS),’ a paediatric patient-reported outcome for spasticity-related pain in cerebral palsy	2014	Questionnaire on Pain caused by Spasticity (QPS)	To report the qualitative development and documentation of content validity for the ‘Questionnaire on Pain caused by Spasticity’ (QPS), a patient-reported outcome and observer-reported outcome for the assessment of spasticity-related pain in children with cerebral palsy	Condition-specific: cerebral palsy	2–16 years
Gilchrist et al. [28]	Development and evaluation of CARIES-QC: a caries-specific measure of quality of life for children	2018	CARIES-QC	To develop and validate a caries-specific measure of quality of life for children that could be used to evaluate different approaches for the management of dental caries. This includes its reliability and responsiveness	Condition-specific: dental caries	5–16 years
Graham et al. [36]	A new measure of health-related quality of life for children: preliminary findings	2007	Child Quality of Life questionnaire (CQOL)	The present study reports the development of a measure that can be used as a generic core for a variety of investigations, involving children with different types of health problem. The study also seeks to determine whether the CQOL is feasible to use in children with different health conditions	Generic quality of life measure	10–14 years
Hareendran et al. [24]	Evaluating functional outcomes in adolescents with attention-deficit/hyperactivity disorder: development and initial testing of a self-report instrument	2015	No abbreviated name mentioned	To identify the impacts of attention-deficit/hyperactivity disorder (ADHD) that are most relevant to adolescents. The study also aimed to explore the feasibility and options available for collecting adolescent self-reports that capture these impacts	Condition-specific measure: ADHD	13–17 years
Hartmaier et al. [70]	Development of a brief 24-h adolescent migraine functioning questionnaire	2001	24-h AMQ	To develop, with adolescent migraineurs, a brief, easily completed measure that would assess the functioning of adolescents during and immediately following a migraine attack	Condition-specific: migraine	11–17 years
Hoffman et al. [52]	Health-related quality-of-life instruments for children with cochlear implants: development of child and parent-proxy measures	2018	CI-QoL	To develop the first cochlear implant-specific health-related quality-of-life measures for school-aged children (6–12 years)	Condition-specific: cochlear implants	6–12 years
Hilliard et al. [51]	Assessing health-related quality of life in children and adolescents with diabetes: development and psychometrics of the Type 1 Diabetes and Life (T1DAL) measures	2019	T1DAL measures for children and adolescents	To design and evaluate the psychometric properties of a suite of developmentally tailored measures of diabetes-specific HRQOL for youth with type 1 diabetes, called ‘Type 1 Diabetes and Life’ (T1DAL). Presented in the paper are two T1DAL measures for children and adolescents	Condition-specific: type 1 diabetes	8–17 years
Khadra et al. [41]	Development of the Adolescent Cancer Suffering Scale	2015	Adolescent Cancer Suffering Scale	To develop a scale to measure suffering in North American adolescents diagnosed with cancer	Condition-specific: cancer	12–18 years
Markham et al. [22]	Children with speech, language and communication needs: their perceptions of their quality of life	2009	No abbreviated name mentioned	This study is part of a programme of research aiming to develop a quantitative measure of quality of life for children with communication needs. The study aimed to provide a qualitative, child-centred description of the quality of life experiences of children and young people with speech language and communication needs	Condition-specific: speech and language issues	6–18 years
Marino et al. [48]	The development of the pediatric cardiac quality of life inventory: a quality of life measure for children and adolescents with heart disease	2008	Pediatric Cardiac Quality of Life Inventory (PCQLI)	To report the development of a disease-specific paediatric cardiac quality-of-life instrument that was generally applicable, and able to discriminate among different types of congenital and acquired heart disease	Condition-specific: cardiac issues	8–18 years
McMillan et al. [40]	The development of a new measure of quality of life for young people with diabetes mellitus: the ADDQoL-Teen	2004	ADDQoL-Teen	This paper describes the design and subsequent psychometric validation of a new teenager-centred, individualised measure of the impact of diabetes on the QoL of teenagers, the ADDQoL-Teen	Condition specific: diabetes mellitus	13–16 years
Morris et al. [47]	Development of the Oxford ankle foot questionnaire: finding out how children are affected by foot and ankle problems	2007	Oxford ankle and foot questionnaire	To use child-centred focus group methods to identify how children’s lives are affected by foot and ankle problems. The issues identified by the children would subsequently be used to generate items for a family-assessed instrument to measure the severity of the foot or ankle problem from a child’s perspective	Condition-specific: foot and ankle problems	5–15 years
Oluboyede et al. [21]	Development and refinement of the WAItE: a new obesity-specific quality of life measure for adolescents	2017	Weight-specific Adolescent Instrument for Economic-evaluation (WAItE)	To report the identification of the final descriptive system of the WAItE, using qualitative interviews with the adolescent population to collect information about the impact of weight on quality of life	Condition-specific: obesity	11–18 years
Panepinto et al. [46]	Development of the PedsQL sickle cell disease module items: qualitative methods	2012	PedsQL Sickle Cell Disease Module	The study reports the qualitative research utilised to develop the new PedsQL™ Sickle Cell Disease Module for paediatric patients with sickle cell disease and support its content validity	Condition-specific: sickle cell disease	2–18 years
Patel et al. [55]	Development of the Malocclusion Impact Questionnaire (MIQ) to measure the oral health-related quality of life of young people with malocclusion: part 1—qualitative inquiry	2016	Malocclusion Impact Questionnaire (MIQ)	To seek the views of adolescents on the aspects of their malocclusion which affect their everyday life and to incorporate these views into a new malocclusion specific questionnaire	Condition-specific: malocclusion	10–16 years
Peterson et al. [31]	Development and pilot-testing of a health-related quality of life generic module for children and adolescents with chronic health conditions: a European perspective	2005	DISABKIDS	To develop a reliable, valid, and sensitive measure to assess HRQOL across health conditions for different countries. The current paper focuses on the pilot testing and psychometric testing of the developed measure—although the initial development steps are also briefly described	Condition-specific: chronic health conditions	8–16 years
Raphael et al. [33]	The Quality of Life Profile Adolescent Version: background, description, and initial validation	1998	Quality of Life Profile: Adolescent Version (QOLPAV)	To report the development of the Quality of Life Profile: Adolescent Version (QOLPAV), including findings from an initial validation provided	Generic: quality-of-life measure	‘High school students’
Ravens-Sieberer et al. [37]	KIDSCREEN-52 quality-of-life measure for children and adolescents	2005	KIDSCREEN-52 HRQOL questionnaire	To provide an overview on the development steps and initial psychometric results of the KIDSCREEN-52 HRQOL questionnaire	Generic: health-related quality-of-life measure	8–18 years
Resnick et al. [39]	Development of a questionnaire to measure quality of life in adolescents with food allergy: the FAQL-teen	2010	FAQL-teen	To create a food allergy-specific quality of life assessment tool explicitly for adolescents in the United States	Condition-specific: food allergies	Adolescents, no specific ages given
Ronen et al. [23]	Health-related quality of life in childhood epilepsy: the results of children’s participation in identifying the components	1999	No abbreviated name mentioned	This paper reports the findings of qualitative research into the different elements of health-related quality of life in childhood epilepsy. The findings are being used to develop a measure of health-related quality of life in childhood epilepsy	Condition-specific: epilepsy	6–12 years
Rutishauser et al. [71]	Development and validation of the Adolescent Asthma Quality of Life questionnaire (AAQoL)	2001	Adolescent Asthma Quality of Life questionnaire (AAQoL)	To report the development and validation of a new asthma-specific health-related quality-of-life questionnaire in adolescents	Condition-specific: asthma	12–17 years
Simeoni et al. [35]	Validation of a French health-related quality of life instrument for adolescents: the VSP-A	2000	VSP-A	To report the major psychometric properties of the VSP-A, including item generation based on the adolescent’s viewpoint, and the testing of these properties	Generic: health-related quality-of-life measure	11–17 years
Stevens [19]	Working with children to develop dimensions for a preference-based, generic, paediatric, health-related quality-of-life measure	2010	CHU-9D	To document the development of relevant dimensions for a new generic paediatric preference-based measure of health-related quality of life	Generic: health-related quality-of-life measure	7–11 years
Tadic et al. [72]	Development of the Functional Vision Questionnaire for Children and Young People with Visual Impairment	2013	FVQ-CYP	The paper reports the development and piloting of a novel instrument, the functional and visual questionnaire for children and young people. Qualitative data from the research program is used to describe children’s own perspectives of what it was like to live with a visual impairment	Condition-specific: visual impairment	10–15 years
Varni et al. [34]	The Paediatric Cancer Quality of Life Inventory (PCQL). Instrument development‚ descriptive statistics‚ and cross-informant variance	1998	Paediatric Cancer Quality of Life Inventory (PCQL)	To describe the item development of the Paediatric Cancer Quality of Life Inventory (PCQL) and to report initial findings on its measurement properties	Condition-specific: cancer	8–18 years
Waters et al. [45]	Development of a condition-specific measure of quality of life for children with cerebral palsy: empirical thematic data reported by parents and children	2005	No abbreviated name mentioned	To identify themes of quality of life for children with cerebral palsy to guide the development of a new condition-specific quality-of-life scale	Condition-specific: cerebral palsy	5–12 years

#### Characteristics of Included Studies

All included studies had a similar aim: to document the development of a measure for children and/or young people. However, the studies differed in terms of how much of a focus there was on reporting the methods for, and results of, the development of the items. Two thirds of the papers discussed the quantitative psychometric validation and development of items, although this was in varying detail, and only seven focused solely on item development. Most studies aimed to develop a condition-specific measure (31/37), with many for use with specific diseases but some also designed for use generically across disease areas, for example, chronic conditions [31, 32]. Six studies reported on the development of generic measures for quality of life or health-related quality of life of CYP [19, 33–37]. Although most studies focused on measuring quality of life in CYP, others also aimed for the measure to be suitable for use in cost-effectiveness analyses and as a preference-based measure [19–21, 32].

Almost two-thirds of the studies used other approaches in addition to qualitative methods to develop items. These studies mostly used literature searches, searches for existing relevant measures and consultations with experts. The exceptions were two studies that used the experience of the research team/authors to decide on the factors important to include [38, 39]. Five of the 22 studies suggested that the findings of these other methods were used to inform the direction of questioning or analysis framework for the qualitative inquiry. However, in most studies these additional methods appeared to be used alongside qualitative methods to either support or add information to the developed items, although it was often not clear how this synthesis of information worked. Two of the 15 studies using qualitative methods only suggested that they thought it optimal for the items to be informed solely by direct research with CYP [19, 23].

Most of the measures reported in the papers had been developed for adolescents (11/37), with the next most common being those developed for CYP aged 0–18 years (6/37) or older primary school-aged children to adolescents (i.e., those aged 8–18 years) (7/37). The remaining measures were developed for primary school-aged CYP aged 5–12 years (4/37), secondary school-aged CYP aged 10–15 years (3/37), all school-aged children aged 5–15 years (*n* = 1) or for use across childhood but excluding very young children aged 0–4 years (3/37). Two papers [33, 39] included unclear information on the age of CYP that their measures had been developed with and for, stating their population as ‘high school students’ and ‘adolescents’ respectively.

Most papers explicitly specified that their measures should only be used with the population that the items had been developed with through empirical work. However, six studies implied that the developed measures could potentially be useful in age groups outside of this. As an example, Varni et al. [34], Ronen et al. [23], McMillan et al. [40] and Gilchrist et al. [28] did not involve any CYP from the upper range of their stated age groups in item development, and Khadra et al. [41] had very little representation from CYP at the lower end. Graham et al. [36] suggested that their measure could potentially be suitable for completion by children (or parent proxies) as young as 5 years, despite the youngest child in their concept elicitation sample being 9 years old. This raises questions around how representative the items in these measures might be for these ‘missing’ age groups, although this is likely to depend on the context and focus of each measure.

Nineteen of the measures involved CYP’s parents/guardians or carers in item development either alongside CYP in paired interviews or focus groups, or in separate data collection. Four papers gave justification for involving parents or guardians, stating that their perspectives can offer additional valuable and valid insight into CYP’s quality of life [24, 42–44]. Others also mentioned practical reasons for involving them—to act as proxies in instances where CYP are not able to participate [20, 43, 45]. One third of the 19 measures involved CYP and parents/guardians separately in data collection where possible, with authors suggesting that this was important to allow CYPs’ individual opinions to emerge [23, 24, 43, 46, 47].

### Overview of Qualitative Approach Used in Children and Young People (CYP) Concept Elicitation

#### Data Collection Methods

The majority (*n* = 21) of included studies used either in-depth or semi-structured qualitative interviews. Eight studies used focus group methods, and six used a combination of interviews and focus groups. One paper used the nominal group technique, where the aim was for participants to present ideas to the group relevant to the factors important to the quality of life of CYP with heart disease [48]. Participants were asked to rank the shared ideas in order of importance. This method differs from focus groups because members do not discuss (the importance of) research themes between themselves, but instead make judgements independently [49]. In the remaining study [33], the methods for data collection were not explicitly stated; however, it was implied that a qualitative approach (most likely focus groups) was used, as the authors described undertaking ‘group meetings’ with high school pupils for instrument development.

Several papers offered justification for their choice of method. Oluboyede et al. [21] discussed using interviews with adolescents to gather individual perspectives on how being obese/overweight affected their quality of life, with the authors suggesting that adolescents felt more confident discussing this on a one-to-one basis. A further four papers suggested that they selected interviews because it either allowed CYP a more comfortable environment to discuss issues, or because it encouraged them to reflect on how their own lives were affected by their condition [19, 24, 35, 36, 38]. Markham et al. [22] and Ronen et al. [23], however, suggested that they used focus groups with CYP because they provided a supportive and social setting that encouraged CYP to share ideas and experiences.

#### The Use of Adapted Data Collection Techniques with CYP

Only five of the 37 papers reported adapting data collection methods to make them more suited to CYP, which for all involved using traditional qualitative methods alongside other techniques designed to involve/engage CYP in research. In the case of Stevens [19], this was setting up a warm-up activity for the children, asking them to decorate name badges to help them to relax prior to being interviewed. The author decided against using props or activities during interviews as they thought it would distract from data collection. However, the remaining four papers used adapted techniques during data collection, including the use of pre-set picture cards [22], drawings [21] and statements [47] aimed at prompting discussion about aspects potentially relevant to CYP’s quality of life. For example, Oluboyede et al. [21] used body shape drawings with adolescent focus groups to encourage participants to consider how individuals with bigger body shapes might be affected by their size.

Two of the papers reported using creative/participatory methods with CYP, asking them to use modelling clay [23] and ‘life maps’ [47] to express ways in which their quality of life is affected by their conditions. In the latter study, CYP were asked to create a character who had a foot or ankle problem and think about and map how that character’s life would be affected by their condition at different times of the day (morning, school, home, weekends). Two studies discussed adapting techniques to the different age groups of CYP [22, 47], with younger CYP in the former study drawing rather than writing about their experiences, and younger children in the latter study taking part in games to select topics for discussion, rather than choosing topics at random as with the older children.

There was suggestion from the studies that those using creative and participatory methods were able to engage their relative CYP population for a longer time period. For example, Markham et al. [22], Morris et al. [47] and Ronen et al. [23] undertook focus groups with those aged as young as 6 years old that lasted from 45 up to 90 min. In contrast, focus groups with 5- to 13-year olds in the study by Gilchrist et al. [28] lasted only 12–14 min. In studies using interviews, Gilchrist et al. [28] carried out interviews lasting 6–16 min, Khadra et al. [41] did interviews with adolescents lasting 18 min on average and Stevens [19]—who used warm up activities with CYP but avoided creative methods during data collection—undertook interviews with 7- to 11-year olds lasting from 4 to 26 min. A summary of the qualitative methods and perceived quality of retrieved papers is in Table 2.

**Table 2:** Details on the qualitative methods and quality of retrieved papers

Paper	Details on qualitative methods	Other methods used for item generation	Details on analysis	Details on sampling	Parent/guardian input?	Quality summary in relation to COREQ checklist
Angeles-Han et al. [65]	Interviews with children with and without vision problems	Interviews with experts and selection of relevant items from existing instruments	No detail on qualitative analysis	No information on sampling	No	Very low detail on qualitative methods. No information provided on sampling, data collection or analysis. Some information on researcher credentials available on title page. No data from interviews presented
Basra et al. [66]	Semi-structured face-to-face interviews with teenage patients aged 12–18 years. Patients were asked to describe ways their lives have been affected by their skin disease	Initial conceptual framework (topic guide) developed from existing literature	Thematic analysis, following grounded theory methodology	Convenience sampling through secondary-referral practice	No	Minimal information on sampling, data collection and analysis. Paper states that saturation reached in relation to themes emerging from interviews. No data from interviews presented
Beusterien et al. [32]	Interviews with children/adolescents with Hunter syndrome and their parents. Interviews were focused on its impact on everyday life	Literature reviews and use of items commonly used in other generic economic measures	States qualitative data analysis used. No further information on analysis approach	No information was given on how children or carers were sampled for the development aspect of the work	Yes, with parents and carers	No information on sampling, data collection or analysis. No presentation of data from interviews. No details given on the credentials of the research team
Bray et al. [20]	Data were collected through face-to-face, qualitative semi-structured interviews in participants’ homes, guided by a piloted interview schedule	The interview schedule was developed from the findings of a previous systematic review, discussion within the research team, and with consideration of the items in existing measures	Framework analysis using an a priori coding framework was used to line-by-line code the transcripts. Codes were grouped into categories of related codes, which were subsequently refined into higher order analytical themes giving a broader understanding of the coded transcripts and the relationship between categories of codes. Child and parent responses were analysed separately	Sampled wheelchair users were stratified by age (0–5, 6–15, 16–18) and by interview set up (child alone, parent alone, parent and child). Potential participants were sent postal information about the study and indicated consent to participate by filling in demographics questionnaire	Yes	Extensive information available on sampling data collection and analysis, although approach to sampling not explicitly outlined. Author provided information on the research team but no reflexivity. Interview schedule and coding framework available. Mentions ethical approval for qualitative study. Authors acknowledge limitations of work i.e. no checking of findings by participants or double coding of data. Uses quotations to support findings
Bruce et al. [42]	Focus groups with children and their families. Questions during focus groups focused on what participants considered important to their health-related quality of life	A literature review of previously generated inventories was used to identify items and dimensions that might be relevant to guide discussions in the focus groups	No formal qualitative analysis discussed	Children approached for participation during routine clinic appointments	Yes	Very little information on data collection and sampling. No information on qualitative analysis or research team or reflexivity. Authors reported reaching saturation in terms of the themes generated from the focus groups
Das et al. [67]	Qualitative study with in-depth interviews and focus groups to inform tool development. Principal caregivers were requested to participate in in-depth interviews, whilst children living with HIV took part in focus group discussions with other children in their age group	A literature review was undertaken, and experts consulted regarding selection of items for the scales	No information on how qualitative data were analysed	Participants recruited with the help of an HIV community-based organisation and through a convenience sample of children with HIV and their caregivers residing in the districts of West Bengal	Yes, with caregivers	Very little information on methods and sampling. No details on research team or qualitative data analysis. No information on how themes were derived from the qualitative data to inform the items of the measure. No presentation of data to support findings
Davis et al. [50]	Interviews were conducted with young people and their primary caregivers. The research used a grounded theory approach, with the interviews aimed at being as open and receptive as possible to allow theory to be developed from the data	No other methods used	The researchers read all responses to identify themes related to quality of life and a list of inductively derived responses was developed. The researchers discussed the interpretation of the data until consensus was achieved. Both researchers re-read the transcripts and coded the patterns by deductively applying the coding framework to each transcript	Families were purposively selected from a hospital register. Families were selected to ensure representation of age, sex and functional severity	Yes	Detailed information available on sampling, data collection and the research team. The authors provided reflection on the impact of their role/characteristics on research findings. Saturation was met and ethical approval for the qualitative research discussed. Information on analysis process not as detailed as other areas, particularly the approach to, and process of, analysis. Use of participant quotes to support findings
Flokstra-de Blok et al. [68]	Adolescents were interviewed on the effect of food allergy on their daily lives	Literature reviews and expert opinion	No information provided on analysis	Participants were recruited from an outpatient paediatric allergy clinic. Two adolescents were approached during a trial, and eight adolescents were approached by telephone	No	Very little information available on data collection, sampling. No information provided on data analysis or the research team. No quotations or themes presented from qualitative data. The paper states that a full description of the methodology is available in unpublished data
Fiume et al. [44]	Interviews explored parent and child perspectives on the impact of child’s stroke on quality of life	Literature review and informal consultation with experts	Qualitative content analysis of interview responses. Based on the analysis, a series of charts were created compiling emergent themes and frequency, and items and domains of concern	No information on sampling	Yes. Adolescent interviews undertaken separately from parents	Very limited information on data collection and analysis. No information on sampling and the research team. No data presented from the interviews to support findings
Follansbee-Junger et al. [43]	Focus groups. Semi-structured, open-ended questions were asked of participants to identify and develop the content of the items. Short interviews undertaken with younger children	Literature review undertaken to generate content and develop the conceptual framework for the focus groups. Expert input	Thematic analysis. Focus group transcripts coded by two separate reviewers. Thematic content examined by three researchers and final decisions on main themes made by consensus	Families recruited during routine medical visits. Sample included spectrum of ages, developmental abilities, sex and type of epilepsy	Yes	Detail included on research team, data collection and analysis. Saturation of interview themes and double coding of data reported. Analysis process not described in detail and no quotes from focus groups data. Findings from interviews with young children not discussed
Franciosi et al. [38]	Focus interviews. All interviewers were trained by an experienced qualitative researcher and provided with a semi-structured interview guide of open-ended questions	A priori domains were developed based on the existing literature and the experience of the research team to inform the measure and questions for the interviews	Responses were grouped according to open-ended questions, domains of interest, and age ranges. Common domain themes were elicited when two or more participants described them, and content themes were then derived by consensus among the research team. Disagreements were resolved by further discussion	Participants were identified from local and referral populations at a hospital medical centre. Children and young people were sampled from different age groups: 5–7, 8–12 and 13–18 years of age	Yes. Children aged 8–18 years interviewed separately from their parent	Good amount of information on researchers’ backgrounds/credentials. Information available on data collection, sampling and analysis. Saturation of themes reported. However, no data reported from the qualitative interviews and no formal qualitative analysis approach stated. Mentions ethical approval for the qualitative study and interview topic guide available
Geister et al. [69]	Paired concept elicitation, semi-structured interviews used, following a topic guide. Initial open-ended questions were used, followed by probing questions on specific symptoms and situations	Current peer-reviewed literature was searched for important concepts to inform the modules of the measure	Content analysis. Interviews were coded using Atlas.ti software. Inter-rater agreement between coders was assessed on approximately 10% of the transcript database. Saturation of concept was determined to have been reached when there were no longer new concepts being coded	Participants and parents/carers identified through patient records and recruited through four diverse clinical sites in the USA. Maximum variation sampling used to recruit children of different ages and severity of conditions	Yes. Paired interviews were conducted with the child and parent or guardian	Description available of sampling, data collection and analysis process. No information on background of research team or reflexivity. Double coding of interview transcripts and saturation of interview themes reported. No presentation of qualitative data to support themes
Gilchrist et al. 2018 and sister paper: Gilchrist et al. 2015 [28, 29]	Focus groups and interviews with children. The focus groups were facilitated by two dentally qualified researchers and took place in a non-clinical room	The interviews were conducted by one researcher and were recorded. The venue and time of the interview were selected by the participant and their family	Framework analysis was used to classify the data according to themes and categories that emerged. Transcripts were analysed independently by two researchers. Recurring themes were identified and then further developed. The themes were then grouped into main and subthemes. Thematic charts were created	Children were purposively sampled from both a primary care dental setting and a dental care service to take part in qualitative focus groups and interviews. Sampling continued until data saturation was reached	No	Most detail on qualitative methods came from sister paper (Gilchrist et al., 2015). Good information from both papers combined on sampling, data collection and analysis. Sister paper covers research team credentials and reflexivity. Double coding of data and saturation mentioned
Graham et al. [36]	Free-ranging, semi-structured interviews were held with parents and some children with chronic physical disorders. Interviews encouraged detail on daily activities to identify how illness had affected these	Existing and relevant quality-of-life measures with both children and adults were reviewed	Qualitative analysis not discussed, only that findings were grouped into themes by members of research team	Sampling for measure item development not discussed	Yes	Very little information on method. No discussion of sampling or analysis for item development. No qualitative data presented to support findings (questionnaire domains). Mentions ethical approval for the qualitative study
Hareendran et al. [24]	Concept elicitation interviews were conducted with adolescents diagnosed with ADHD and their primary caregivers. A conceptual framework was used to inform the structure and content of the concept elicitation interview guide. Interviews started with an open-ended discussion about the impact of ADHD, followed by questions on specific issues	Literature review and expert interviews to inform conceptual framework for interviews	A content analysis approach was used to analyse data from the interviewers’ field notes, and from the transcripts of audio-recorded interviews. A coding dictionary was developed based on the themes and concepts that emerged during the discussions. Analysis was conducted by two of the authors	Participants were recruited from seven clinical sites from different regions in the USA. A purposive sampling method was used to recruit the sample	Yes, primary caregivers	Good level of information available on sampling, data collection and analysis. No information on research team or reflexivity. Saturation and double coding of interview data reported. Themes supported with quotations from qualitative data, but no topic guide supplied. Ethical approval mentioned
Hartmaier et al. [70]	Unstructured in-depth interviews with 10 adolescent migraineurs	Literature reviews and interviews with experts	No discussion of how interview findings were analysed	Ten adolescent subjects with migraine who were considered articulate about their migraine experiences were recruited	No	Minimal detail on qualitative method used. No discussions of sampling or data analysis. No background provided on the research team. Ethical approval and informed consent for study participation discussed
Hilliard et al. [51]	Individual qualitative interviews using semi-structured interview scripts. The researchers asked open-ended questions and used prompts and probes to elicit additional comments or clarify responses. Interviewers also invited participants to discuss any other topics related to type 1 diabetes that they felt were important	Review of existing relevant instruments and literature to allow study team to generate a preliminary list of health-related quality-of-life topics for potential inclusion as items in the measures	No formal qualitative analysis discussed. The interviewers audio-recorded the interviews and recordings were transcribed. The study team then designed the items to reflect the themes from the qualitative interviews and previous literature. Expert collaborators reviewed the draft measures and provided feedback	Study staff reviewed patient schedules to identify eligible youth with upcoming medical appointments and sent study information letters via email, followed by a telephone call to introduce the study and schedule a visit	Yes. Youth interviews conducted separately from parents	Good information available on data collection, sampling and examples of interview questions. Mention of ethical approval for item development and consent/assent from participants (including youth) for the qualitative study. However, no mention of any formal qualitative data analysis and no quotations to support themes from the data. Some reference to researcher background characteristics
Hoffman et al. [52]	Qualitative interviews with children with cochlear implants and their parents. Discussion guides included an outline of open-ended questions and a series of follow-up probes to elicit additional information	A literature review and focus groups with stakeholders were used to create conceptual framework that was followed during interviews with children	Content analysis. To identify common themes and generate initial codebooks for the coding tree, transcripts were randomly selected. The authors grouped phrases from the transcripts by theme to create codebooks and these were then used to code all transcripts. Transcripts were coded in pairs to achieve consensus coding	To ensure demographic and geographical diversity, children were recruited from national, paediatric cochlear implant centres. Flyers about the study were distributed to all families of paediatric patients in the desired age range	Yes	Some information available on data collection and sampling. Detailed description of analysis process and presentation of some quotations from interviews. Diagram of conceptual coding framework given. Ethical approval for the study and saturation of content from interviews mentioned. No information on research team or reflexivity
Khadra et al. [41]	Interviews were conducted with individual adolescents in a private office at the clinic or in the patient’s room. The interviews were semi-structured, based on a list of open-ended questions	The conceptual model used to guide the content of the semi-structured interviews was based on the components of an existing quality-of-life model for cancer survivors	The Corbin and Strauss method of constant comparison, including immersion, coding, categorisation and grouping was used to analyse the content of the interviews. Authors examined the data and contextual references and searched for differing meanings of words. Line-by-line analysis was performed to assign appropriate codes to units of data	Convenience sampling method used for recruitment through an outpatient clinic in a paediatric hospital	No	Information available on sampling, data collection and analysis. Some information given on research team and their role in research. No discussion of saturation or presentation of quotations from interview findings. Ethics and informed consent for study participation mentioned
Markham et al. [22]	Focus groups with children and young people including the use of enabling techniques, which provided participants with additional and alternative methods of exploring and responding to research questions, including the use of a picture-card game designed to encourage children to relate their own experiences during discussions	No other methods used	Analysis used grounded theory and framework analysis. Transcripts were searched for units of meaning relating to the research question. These units were indexed with descriptive labels in a process of open coding using constant comparison analysis. As analysis progressed and new codes were added to the index, these were also iteratively applied to transcripts previously analysed	All participants included in the study were aged between 6 and 18 years; attending full-time education within a mainstream education setting, including language units, or a special school for children and young people	No	Detail on qualitative method and enabling techniques and analysis. Limited information on sampling. Discusses reflexivity and how background of the researcher may have influenced findings. Mentioned reaching saturation of focus group themes. Ethics and informed consent discussed
Marino et al. [48]	Data collection used the nominal group technique, where members respond to a set of scripted questions, after which a single idea is put forward by each participant until all ideas generated from the scripted questions are discussed. The scripted questions focused on issues important to children’s/adolescents’ quality of life with heart disease	No other methods used	Ideas from the nominal groups were entered into a cumulative list of potential items. Items on the cumulative master lists were then separated into a priori hypothesised dimensions. The research team met to review content, and through note summarisation and constant comparison deleted redundancies within cumulative lists	Potential nominal group members were identified through the Cardiac Center database at a children’s hospital. Eligible patients were sorted alphabetically, and every third patient/parent was contacted. Eligible patients were invited by telephone to participate	Yes	Processes of sampling, data collection and analysis discussed. Ethical approval and informed consent for participants mentioned. Some details on research team given. Some examples of questions asked to groups given in text. No quotations from nominal group data to support findings
McMillan et al. [40]	Semi-structured interviews using open-ended questions were conducted with teenagers with diabetes, and focus group discussions took place with teenagers in small groups of 2–4 teenagers each	No other methods used	No information on qualitative analysis	Participants were sampled from four hospitals in Greater London	No	Lack of detailed information on sampling and data collection. No information at all on qualitative analysis. Limited information on sample of teenagers participating in research. No quotations from interviews/focus groups to support research findings. No information on research team. Ethical approval for study mentioned
Morris et al. [47]	Focus groups. Each group was led by a facilitator experienced in conducting focus groups with children. In the first session, participants were invited to agree or disagree with the pre-set statements regarding their quality of life with a foot or ankle problem. The second activity involved life-mapping, in which the groups were asked to consider issues arising during a day in the life of a child with a foot or ankle problem	No other methods used	The audio recordings were transcribed, and the accuracy checked. Grounded theory and content analysis were used to group each of the issues that participants had raised. Each part of the transcripts was coded by comparing the text with pre-set constructs and the comments provided by others. The verbatim statements were subsequently aggregated into categories and labelled accordingly	Children using health services for foot and ankle problems were identified by healthcare professionals at an NHS orthopaedic hospital. The families of those children who were between 5 and 15 years old and had attended the hospital in the preceding 2 months were mailed invitations to take part in a focus group	Yes, involved in separate focus groups	High level of detail available on data collection. Some information on sampling and analysis. Extensive quotations from focus groups reported to support findings. Ethics approval for the research mentioned. Limited information on research team
Oluboyede et al. [21]	One-to-one interviews conducted to gather information on how being overweight impacts aspects of life. Focus groups with treatment seeking and non-treatment seeking adolescents for wider views on issues of importance	Review of existing weight-specific instruments to guide topics of questioning during interviews	Framework analysis. Themes were identified from listening to interviewed recordings and reading through transcripts using an iterative process. A matrix summarised and synthesised data generated from the interviews	Adolescents recruited from three UK-based weight management centres and one school. Sampled purposively according to gender and age	No	Information on sampling, data collection and analysis. Topic guide for interviews provided. Analysis validated by a second reviewer. No qualitative data (quotes) presented to support findings. Very little information given on research team
Patel et al. [55]	Interviews. Open questions to avoid leading participants’ answers. Topic guide developed and used in a flexible manner and adapted as data collection progressed. Interviews were split between a non-clinical environment and participants’ homes	Prior to carrying out the interviews, a topic guide was developed with reference to existing literature	Framework analysis. Transcripts from interviews were read and notes were made independently by the two interviewers on the general themes emerging. An initial thematic framework was developed and discussed within the study team. Sections of transcripts were labelled by the interviewers to indicate which themes data related to. Thematic charts were created for the main themes	Potential participants were identified by the clinician treating them in the orthodontic departments at two National Health Service (NHS) hospital Trusts. Purposive sampling was used to ensure representation of key characteristics: age, gender, ethnicity and malocclusion type	No	Detail available on sampling, data collection and analysis. Some details given on research team i.e. researcher backgrounds and qualifications. Presentation of quotations from data to support themes and item development. No presentation of topic guide or coding framework
Panepinto et al. [46]	In-depth interviews conducted with paediatric patients with sickle cell disease. Open and semi-structured questioning was used to elicit themes around issues identified as important from the literature review and experts	A literature review was undertaken to identify important issues for the interviews. Expert opinion was used to review the domains	A content analysis was performed. Attention was paid to the frequency, extensiveness, specificity and emotion of the themes. Themes were later grouped into appropriate disease and treatment-related areas to inform domains	Participants were recruited from a disease-specific clinic in the US. Purposive sampling ensured that different age groups and clinical phenotypes were represented	Yes. Separate parent and child interview undertaken. Children aged 5–7 years were interviewed with parent present	Saturation mentioned. Analysis performed by three researchers. Authors gave background information on the research team. Interview topic guide included in paper. No information on how participants were approached for participation. Presentation of some quotes from interviews
Peterson et al. [31]	Focus groups with children and adolescents. At the beginning of groups, questions were asked about how they view their condition and how they cope with it	Literature review of other health-related quality-of-life measures to inform measure development	Statements from the focus groups were grouped into three sections to inform the measure: (a) generic (b) chronic generic and (c) condition specific	No information on sampling apart from that the focus groups were stratified by age and severity of disease	Yes	Very little information on sampling and no formal qualitative analysis reported. No presentation of themes from the focus groups. No information on the research team
Raphael et al. [33]	Authors suggest that focus groups methods were used but this is not made clear. “Instrument development began with a series of six group meetings with high school students.” Adolescents were asked what the term “quality of life” meant to them	Adolescent development and adolescent health literature were drawn on in item development	Responses from the participants were collected, reviewed by the authors, and developed into instrument items	No information on sampling	Yes	Not clear whether formal qualitative research method used. Limited information on sampling and data collection. No mention of formal qualitative analysis. No presentation of data to support focus group themes. No information available on research team
Ravens-Sieberer et al. [37]	Focus groups with children and adolescents discussed different aspects of their perceived quality of life. Facilitators followed a protocol which contained open questions to very narrow questions	Literature reviews and expert consultation (Delphi study) were used alongside focus groups to determine the dimensions of the measure	No discussion of formal qualitative analysis. Statements derived from the focus groups were rewritten into an item format and reduced using quantitative techniques (including card sorting techniques)	Focus groups took place across different country settings, with children and adolescents of different age ranges and gender	Yes. Parents of children and adolescents were included in the focus groups	Very limited information on sampling and data collection. No discussion of nature of parental involvement in focus groups. No formal qualitative analysis mentioned. No presentation of quotations from focus group data. No information on research team
Resnick et al. [39]	Focus groups with food-allergic adolescents	Information from literature reviews and the experience of the authors were used alongside focus groups to develop questionnaire items	No formal qualitative analysis discussed	No information on sampling aside from that focus groups took place across three states in the USA	No	No information on sampling, analysis or the research team. No information on how focus groups were conducted or on characteristics of the adolescents involved. No presentation of data from focus groups. Does mention ethical approval for study
Ronen et al. [23]	Focus groups with children with epilepsy. The groups were modified with pre-set activities to prompt the discussions, which were facilitated by child-life specialists. Activities included drawing maps of important places in the child’s daily life to elicit discussions about their external world and forming playdough to trigger dialogues about their internal world. Each group discussion lasted 90 minutes	No other methods used	Textual analysis of the raw data using the Ethnograph V4.0 software. This consisted of identifying the components of health-related quality of life. The process of coding, categorising, and reassembling the raw data was continuously revised as the field work continued. A higher level of textual analysis followed, discovering relationships and trends, and clustering the codes into smaller numbers of dimensions	Stratified purposeful sampling. Children registered on the Child and Adolescent Epilepsy Database were approached for the study. Families who met the entry criteria were invited by a letter, followed up by a telephone call, to participate in the focus groups. Children were stratified by age and duration of epilepsy	Yes. Parents participated in separate focus groups	High level of information on sampling and data analysis. Focus groups findings were validated with a subset of the original participants. Saturation of the categories emerging from the focus groups was reached. Double coding of focus group data undertaken. No information on research team. Adapted methods were used with children during focus groups. Participant quotations available to support findings
Rutishauser et al. [71]	Focus groups and three single interviews were used and began with open-ended questions followed by semi-structured interview questions	An initial pool of items for item selection were generated by a critical review of the literature including existing health-related quality-of-life measures and expert opinion	No formal qualitative analysis discussed	Participants were recruited from paediatric asthma clinics in two tertiary hospitals	No	No information on how qualitative data were analysed. Limited information on data collection and sampling. No presentation of quotations from qualitative data. Saturation mentioned. No information available on research team
Simeoni et al. [35]	Interviews with adolescents. The first part of the interviews were conducted by a trained interviewer and explored in a nondirective way the impact of health on their quality of life. The second part was a semi-structured interview concerning principal topics reported in the international literature	Results of literature review used to inform topics explored in the interviews	Interviews were recorded, transcribed and analysed using content analysis	Adolescents attending public schools in a south-eastern county of France were randomly selected. The population was stratified according to age and socioeconomic status	No	Saturation reached with interview data. Very little information on sampling, data collection or analysis. No empirical data presented from interviews. No information available on the research team, and very little on sampled adolescents
Stevens [19]	Semi-structured interviews to ask children about how health problems affected their lives. A topic guide was used to facilitate probing around important issues. The format of the interview was first to ask the child about any health problems and then ask additional questions about how the child’s health affected her or his life. All questions asked were open-ended	The author used qualitative research only to ensure that existing literature and measures would not influence findings and item selection	Thematic content analysis with Framework analysis used to identify dimensions of health-related quality of life directly from the data. Data were analysed using NVivo software. The data were charted, producing a matrix of subthemes and respondents. Each subtheme was reviewed for explanations behind the affected areas of health-related quality of life to develop the dimensions	Purposive sampling. Children were sampled from two schools in Sheffield, UK. Schools were chosen to represent diversity in ethnicity and social class. Children were also sampled according to age and health status	No	Sampling, data collection and analysis described in good level of detail. Saturation reached. Quotations from interviews included in the paper to support themes. Author gives some information on background and possible biases (reflexivity). Data only coded by one researcher (although analysis overseen by another researcher). Length of some interviews very short (4–26 minutes). Mentions ethical approval and informed consent (assent)
Tadic et al. [72]	Semi structured interviews. Questionnaire items were developed by grouping qualitative statements related to general activities, activities related to visual impairment, level of functioning, restrictions and limitations in activities and mobility	Themes from an existing measure were used to inform the analysis framework for qualitative interview data (home, school and leisure themes)	Two researchers independently coded interviews using Nvivo 9 software, grouping together all relevant statements. Statements were reviewed by another two researchers who rated all the statements, with these ratings being compared to inform the final item pool	Stratified sampling approach used. Databases of eligible patients attending two eye hospitals and clinics in the UK were recruited	No	No formal qualitative analysis described. No information on the research team. Some information available on data collection and sampling. Multiple researchers coding qualitative data. Ethics approval for the study mentioned. No themes for interviews presented
Varni et al. [34]	Open-ended interviews with children and their families	Questionnaire initially based on an extensive search of the relevant literature and discussions with healthcare professionals who care for paediatric cancer patients	No information on qualitative analysis included	Participants were recruited at three major paediatric cancer centres. Description of the sample is also provided (inclusion and exclusion criteria)	Yes. Interviews with parents also	No discussion or detail on data collection, analysis or research team. Very little information on sampling. No presentation of qualitative data from interviews. No information on how parent interviews were carried out
Waters et al. [45]	Interviews with families of children with cerebral palsy. Interviews lasted 30 minutes	Interview questions were derived from a review of the quality of life literature	The study employed a grounded theory approach. Themes from the interviews were extracted by three researchers. Agreement on key themes was achieved by discussion	Purposive sampling. Families were selected from the Victorian cerebral palsy register (maintained in Melbourne). The sample was intended to be representative of age, socio-economic status, functional severity and geographical location	Yes. Mostly parental interviews but some children with mild impairments were able to take part in interviews with their parents present	No discussion of research team or suggestion that any formal approach to qualitative analysis was used. Topic guide available in appendix of paper but no presentation of interview data. Mentions ethical approval for the study. Some information on aspects of sampling and data collection

### The Quality of Reporting in Concept Elicitation for CYP

The retrieved papers varied in terms of the number of COREQ checklist criteria met; however, almost half of the papers reported on none or very few of the 32 quality indicators.

#### Reporting on Data Analysis

Papers tended to miss reporting information on data analysis, with 15/37 not including any information on the approach to qualitative analysis used. An additional four papers included only very brief information on analysis, including the technique used (e.g., content analysis or constant comparison) but with little or no information on the process of data analysis, that is, how codes were developed and applied to the data and how themes were identified. In terms of findings, only eight of the 37 papers included quotations from the data to support the themes that had informed the items of their measures.

#### Reporting on Sampling

Seven papers included no information on sampling at all. A further seven studies included very basic information on either the sampling strategy (e.g., convenience or purposive sampling) or where participants were identified. The papers generally lacked information on the methods for initially contacting participants (e.g., though face-to-face consultation or postal invite) and information on those who had declined to participate. Two papers also lacked basic information on the age of the CYP included in their study [33, 39].

#### Reporting on Data Collection

More information was generally available on data collection, with all but one paper [33] making clear which data collection method they had used. Just under one third of the papers gave an indication of the average duration of focus groups or interviews, and a similar number mentioned reaching saturation of the themes identified to inform items. However, only nine papers included an interview/focus group topic guide or examples of the questions that were asked to participants. The papers also tended not to include information on where data collection took place and who was present.

#### Reporting on Research Team and Reflexivity

The most common area in which information was lacking was on research team and reflexivity, with only eight [22, 28, 38, 41, 43, 46, 50, 51] of the 37 papers including any sort of background information on the researchers (including gender and academic background). Of these seven papers, only two provided reflections on how the backgrounds of the authors may have influenced data collection or the nature of research findings. For example, Gilchrist et al. [28] commented on the potential impact of the researcher’s role as a dentist when exploring the consequences of dental caries on children’s quality of life. The authors reflected that due to the researcher not being the children’s personal dentist, it would have been unlikely to have inhibited children’s interview responses—and further, because the researcher was not aware of the children’s dental history until after interviews had been undertaken and transcripts analysed, it was unlikely to have affected the nature of this researcher’s questioning or analysis. In contrast, Davis et al. [50] reported that the comprehensiveness of their findings on the impact of cerebral palsy on adolescents may have been impacted by both the researchers being female, with the possibility that male adolescent participants may not have felt comfortable discussing more sensitive issues (such as relationships) with female researchers. Markham et al. [22] acknowledged that his professional and academic background would have potentially biased data collection and analysis but suggested that this potential had been “mitigated by the facilitator’s reflexivity, whereby a priori preconceptions were consciously noted and attempted to be bracketed from the study” [p. 753]. However, the author gave no indication of what these biases might have been, and how they had been avoided.

#### Strengths in Reporting

Despite many of the papers meeting limited quality criteria on the COREQ checklist, there were strengths to some of the studies reviewed. Eleven met 15 or more of the 32 checklist criteria, including greater coverage of information on sampling, data collection and analysis than other papers. Four studies (three of these being those identified as meeting a high number of criteria on the COREQ) reported following FDA guidelines for measure development [19, 21, 46, 52] and a further study (also highly detailed) mentioned following the COREQ guidelines for reporting [20]. Twenty of the 37 papers stated that they had ethical approval for the qualitative study, with twelve mentioning gaining informed consent (or assent) from research participants. It is important for researchers to show that they have thought about ethical issues, particularly when conducting research with CYP who may be vulnerable to pressure to take part in studies or who may not fully understand what they are being invited to participate in [53, 54]. However, despite the acknowledgement of ethical procedures within many of the papers, only two of these mentioned developing study information sheets specifically for CYP’s understanding, which if not developed, may have limited CYP’s ability to give informed assent for their participation in research [14].

## Discussion

The review retrieved a total of 37 papers, featuring condition-specific and generic measures to record changes in the quality of life of CYP. Most studies had developed measures for adolescent populations and had used either interviews or focus groups for item generation, with those choosing interviews seemingly because the method provided a more comfortable environment for CYP to discuss individual and potentially sensitive issues. This fits with previous recommendations made for PROM development in paediatric populations, which suggest that focus groups might lead to social desirability bias, as CYP could feel inhibited to express their own opinions and more likely to agree with previously raised themes in group situations [11]. Therefore, the use of focus groups in this context could potentially cause problems around the representation of all CYP’s views in item generation. However, similar issues could conceivably arise in interviews, in situations where CYP might feel compelled to answer questions in a manner that they think will be viewed favourably by the interviewer.

A relatively low number of studies discussed adapting methods to be more suitable for the CYP population, with only four using creative and participatory methods alongside interviews and focus groups. Several PROM guidance papers recommend the use of such approaches with CYP to keep their attention [11] and to help overcome anxiety and encourage discussion [55]. Further, studies in the child methodology literature recommend these methods to allow CYP more time and freedom to express themselves, and to address power imbalances between CYP and adult researchers, by giving CYP more control over the topic and direction of research [12, 13, 56, 57]. Those using creative and participatory methods in the studies collected here appeared to engage their CYP population for a longer period, and although length of data collection is not necessarily an indication of quality, relatively short data collection periods might suggest that aspects important to a population may not have been discussed fully or in depth. The suggestion from the literature and this review therefore is that participatory and creative methods can be beneficial in helping CYP to engage in concept elicitation work in a more meaningful way, potentially helping to enhance the coverage and validity of included items.

However, the literature suggests that these methods are particularly relevant for engaging and keeping the attention of younger age groups [11, 55], with Arbuckle and Abetz-Webb recommending the use of creative approaches in research with 6- to 11-year olds, with traditional qualitative methods becoming more appropriate in adolescents aged 12 years and over [11]. Indeed, several studies in this review appeared to carry out successful concept elicitation work with very young children (as young as 6 years), and the increased use of such methods in this area may help with the development of further measures for younger children, which at the moment are less common than those for adolescents.

In terms of reporting quality, although there were strengths, none of the 37 papers met all criteria outlined on the COREQ checklist for qualitative research, and almost half of the papers met two, one or zero. Further, many of those meeting criteria did so in very little detail. Detail was most lacking on qualitative data analysis, sampling and the research team, with these missing details making it difficult for the reader/user to make judgements about content validity and whether the items in the measures had achieved full coverage. For example, evidence of a robust sampling strategy is crucial in ensuring that important characteristics of a population have been captured (i.e., purposive sampling) [58] and, in several of the studies retrieved in the review, there was no representation in the empirical work from specific age groups within their stated population. This is particularly important in light of guidance from the FDA and others [5, 11], which state that measures should be developed and saturation of items achieved in narrow age groupings of CYP, due to the rapid changes that take place in their developmental and cognitive abilities during childhood and into adulthood [59].

Details on the processes of qualitative data analysis and the research team are important to allow judgements around the robustness of the authors’ interpretations of collected data. Reflexivity regarding the authors’ acknowledgement of how their own personal characteristics and assumptions may have influenced findings is essential to judgements around validity [9, 60] and this review found that only a small number of papers had disclosed and discussed this information. Qualitative quality guidance states that researchers should be explicit about how final themes and concepts are developed from data and provide evidence in quotations from participants to support these [27]. This review has demonstrated that very few studies had a high level of detail on the analysis process, and under a quarter of the retrieved studies included any quotations to support the items generated, leaving measure content without a clear evidence base.

Many studies used other methods with qualitative data collection to inform measure items, such as literature reviews, expert opinion and even the expertise of the authors. Although these are potentially valuable sources of information [7], it is ambiguous in many of these papers as to how far final measure content was informed by CYP’s own opinions and experiences of what is important. An important quality indicator is transparency in the reporting of research processes and how research conclusions are generated [61] and this review has indicated that reporting of qualitative concept elicitation for CYP measures appears to be generally lacking in this respect. This mirrors findings of a systematic review of condition-specific preference-based measures (PBMs) by Brazier et al. [62], who found that measures using qualitative analysis in item development had reported their methods in very little detail, with the authors describing this as a ‘barrier’ to this aspect of measure development being better understood and becoming more scientifically rigorous (p. 26–8).

To the authors’ knowledge, this is the first review to summarise and critically analyse the qualitative methods used for concept elicitation for measures for children and young people. Existing reviews of generic paediatric measures have tended to summarise and critically analyse the items contained within the measures (e.g. [17, 18]) or review the usage of the measures in practice (e.g. [16]), with condition-specific measure reviews tending to summarise the measures available in particular disease areas. The strength of this review is that it has focused on how researchers have reported concept elicitation with CYP [5, 7], and has importantly highlighted where more transparency is needed to allow judgements around content validity. Although research teams are clearly recognising the value of having direct input from CYP into item development, the poor quality of reporting in these studies raises questions around how far the content of these measures is truly sensitive to what is important to these populations.

Despite this review critiquing the quality of reporting for concept elicitation in CYP measures, it is important to note that it is not necessarily that researchers have not followed robust research processes, but that this has not been made clear and described in a high level of detail. For example, some of the research teams also went on to perform further validation tests with CYP on the developed items, which may have strengthened content validity (i.e., using qualitative cognitive interviews with the relevant population to check their coverage). It is also important to acknowledge that these studies have followed recommendations to use qualitative methods in item generation. Given that the focus of this review has only been to retrieve studies using qualitative methods for concept elicitation, we are unable to calculate the number of studies not using qualitative research, but we know that in economics, for example, the vast majority of PBMs for child economic evaluation have not included CYP in item development [16]. The measures included here have therefore been successful in facilitating the inclusion of the ‘patient voice’ in content development, which is particularly important given that children and young people have often been excluded from research [63].

This review only searched for papers in peer-reviewed journals and it is possible that further papers may have been retrieved if the grey literature had also been searched. Further, a few more relevant papers may have been picked up if the search terms had been expanded slightly—for example, to include ‘health measures’ in the ‘focus and outcomes of developed measures’ criterion of the search. However, the authors used additional techniques such as searching in relevant systematic reviews and forward citation and reference list searching to encourage a more comprehensive and targeted search. It is unlikely that the inclusion of additional studies would have changed the overall message of this review, as the reporting quality was low or lacking in most included studies. It is possible that the authors of this review could have contacted the authors of the retrieved studies for further information on concept elicitation, but in practice this would not be helpful to the users of measures who need to make judgements around content validity using the (published) information that is readily available to them. Having said this, it is also important to note that authors are often restricted by manuscript length limits and the need to report other aspects of measure development. The development of detailed guidelines on how to undertake qualitative concept elicitation work with CYP [7], and particularly on what to prioritise when reporting measure development, may help to overcome issues around poor reporting and content validity, and therefore should be considered an important area for future research.

## Conclusion

This systematic review has summarised the qualitative methods and, where relevant, the adapted data collection techniques used to develop the conceptual items in measures for children and young people. We found that very few of the retrieved studies had used creative and participatory methods for item development, despite these approaches being potentially beneficial for engaging children and generating more meaningful data for concept elicitation, particularly with younger populations. The review identified important gaps in terms of the quality and transparency of reporting for item generation, with many studies not reporting information central to establishing content validity. This review recommends that research teams report concept elicitation work with children and young people in greater detail, with the development of methodological and reporting guidelines in this area being key to facilitating this.

## Electronic supplementary material

Below is the link to the electronic supplementary material.

Supplementary material 1 (DOCX 11 kb)

## Data Availability

Data sharing is not applicable to this article as no datasets were generated or analysed during the current study.
