# Supplementary material for: A Systematic Review of the Use and Quality of Qualitative Methods in Concept Elicitation for Measures with Children and Young People
Source: Patient. 2020 Apr 29;13(3):257–88. doi: 10.1007/s40271-020-00414-x (PMC7210227; doi:10.1007/s40271-020-00414-x)
Supplement: Supplementary file 1 — Supplementary material 1 (DOCX 11 kb) [file 40271_2020_414_MOESM1_ESM.docx]

**Appendix 1: Search terms developed for use in electronic databases**

| **Focus of term** | **Search terms used** |
| --- | --- |
| Population | “Children” OR “young people” |
|  | **AND** |
| Methodological approach to measure development | “Qualitative methods” OR “qualitative methodology” OR “qualitative” |
|  | **AND** |
| Focus and outcomes of developed measures | “Economic measure development” OR “quality of life measure development” OR “capability well-being measure development” |

**Appendix 2: Data extraction form**

**[Authors and title of paper]**

**Paper details:**

Title:

Author:

Year:

Name of measure:

Method of retrieval:

**Study and measure characteristics:**

Objective of study:

Type of measure (generic or condition specific):

Age of children/young people measured developed for:

Parents/guardians (dyads) involved in measure development?

**Information available on qualitative methods:**

Qualitative methods used:

Adapted methods used:

Qualitative analysis:

Sampling:

Additional material available (e.g. topic guides?):

Positive reflections on the method (authors’ and research teams’):

Reported limitations of the method (authors’ and research teams’):

**Other information relevant to the review**

Other methods used for item development (aside from qualitative):

Any other information relevant to the review question:
